#  Genetic markers of the risk of coronary heart disease and coronary artery thrombosis developing in the Kazakh population

**DOI:** 10.22088/cjim.14.2.249

**Published:** 2023

**Authors:** Dana Taizhanova, Aliya Toleuova, Dmitry Babenko, Anar Turmuhambetova, Roza Bodaubay, Olga Visternichan, Akerke Kalimbetova, Ludmila Ahmaltdinova, Aigul Kurmanova

**Affiliations:** 1 NJSC "Medical University of Karaganda", Karaganda, Kazakhstan

**Keywords:** gene polymorphism, coronary heart disease, coronary artery thrombosis, coronary artery restenosis, percutaneous coronary angioplasty, Kazakh population.

## Abstract

**Background:**

Surgical methods such as coronary artery bypass grafting and percutaneous coronary interventions (PCI) are widely used along with traditional conservative therapy in the treatment of coronary artery disease. The disease outcome directly depends on timely diagnosis and treatment. A significant role in predicting the effectiveness of treatment is given to personification of treatment and management of the patient. In this case, the determining component is its individual genetic status.

**Methods:**

The study groups included persons of Kazakh nationality which identify themselves, their biological parents, and biological grandparents on the maternal and paternal side as Kazakh. Research groups included 108 people at the age from 45 to 65 years of both sexes. Blood samples genotyping was carried out by PCR using highly specific TaqMan samples. Thermo Fisher cloud application was used for genotypes determining on the base of an automatic algorithm.

**Results:**

The article presents the results of the evaluation of gene polymorphisms associated with coronary artery restenosis in a population of Kazakh nationality. 3 SNPs were determined when searching for an association with stenting due to coronary artery thrombosis: rs7543130 (p=0.009324), rs6785930 (p=0.016858), rs7819412 (p=0.061325).

**Conclusion:**

Four polymorphisms associated with the risk of developing coronary heart disease were revealed during the study of polymorphisms among the people of the Kazakh population. Three SNPs were determined when searching for an association with stenting due to coronary artery thrombosis. It should be noted that the Bonferonni correction for multiple comparisons did not reveal significant polymorphisms associated with coronary artery disease, which requires further research with more quantity of samples.

Coronary heart disease (CHD) is one of the main causes of cardiovascular mortality (1). Despite significant progress in the development and application of modern methods of diagnosis and treatment, diseases of the circulatory system are the most important problem in terms of mortality in the Republic of Kazakhstan (2). According to WHO experts, over the past 10 years there has been a tendency to increase the number of diseases of the circulatory system by 1.3 times (from 1984.4 per 100 thousand population in 2003 to 2523.0 per 100 thousand population in 2012) (3). In general, diseases of the cardiovascular system in Kazakhstan are the cause of almost 1/3 of all deaths. In the structure of mortality from diseases of the circulatory system 34% are patients with coronary heart disease, of which more than 30% are persons of active working age (18-65 years old) (4,5). 

Surgical methods such as coronary artery bypass grafting and percutaneous coronary interventions (PCI) are widely used along with traditional conservative therapy in the treatment of coronary artery disease. The disease outcome directly depends on timely diagnosis and treatment. A significant role in predicting the effectiveness of treatment is given to personification of treatment and management of the patient. In this case, the determining component is its individual genetic status. To evaluate the genetic polymorphisms determining the predisposition to coronary heart disease in individuals of the Kazakh population and the predisposition to subsequent coronary artery thrombosis, that necessitated stenting.

## Methods

The study groups included persons of Kazakh nationality which identify themselves, their biological parents, and biological grandparents on the maternal and paternal side as Kazakh. Permission of the Committee on Bioethics of KSMU No. 305 dated May 19, 2017, was received for conducting research. The subjects were divided into the following groups: the I^st^ group – 58 people at the age from 45 to 65 years of both sexes. Inclusion criteria: patients with a diagnosis of coronary heart disease receiving traditional medication based on the recommendation of the European Society of Cardiology (ESC). The II^nd^ group – 58 people aged from 45 to 65 years of both sexes. Inclusion criteria: patients with diagnosed coronary artery disease after stenting due to coronary artery thrombosis and lack of restenosis during the year (ESC). The control group included 50 people at the age from 45 to 65 years of both sexes. Inclusion criteria: practically healthy persons (not registered in the dispensary). A survey of patients was conducted to identify risk factors for thrombosis, gender and anthropometric characteristics (age, height, body mass index, waist circumference) were assessed, a detailed analysis of the provided medical documentation of patients was carried out - discharge summaries (based on the written consent of the patients). The study material was the blood of patients and conditionally healthy people. Blood sampling was carried out as standard in the morning, on an empty stomach. 2 ml of whole blood was taken into a vacuum container with the anticoagulant EDTA for molecular genetic studies. Blood for molecular genetic studies was collected once. 

**Figure 1:**
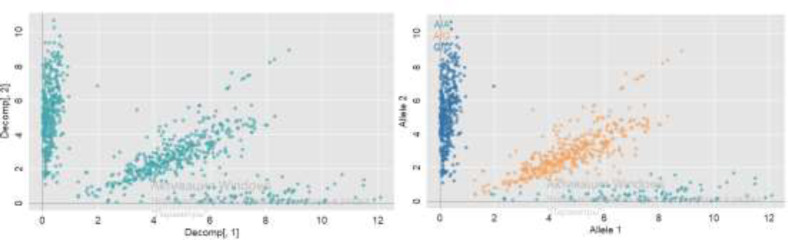
Two-dimensional distribution of genotyping results

Total whole blood DNA was extradited using the commercial GeneJET Genomic DNA Purification Kit (Thermo Scientific). The concentration and degree of purification of the isolated DNA were monitored spectrophotometrically using a P330 nanospectrophotometer (Implen) and a LabChip chip electrophoresis system. Genotyping of samples was carried out using QuantStudio TM 12K Flex Real-Time PCR (Applied Biosystems) technology using genetic panel (55 polymorphisms in each). The analysis of PCR results (QuantStudio 12K) was carried out in the ThermoFisher cloud service (https://apps.thermofisher.com). The results of each PCR reaction were presented in a 2-dimensional space to visually assess the distribution into pools (figure 1). After automatic annotation and visual inspection, genotypes were determined for each sample (patient). Figure 1 - Two-dimensional distribution of genotyping results The following indices were determined for each polymorphism included in the genotyping panel: major and minor alleles, an indicator of minor allele frequency (MAF – minor allele frequency), relative values for alleles and genotypes, and also an indicator of p-value when calculating the Hardy – Weinberg equilibrium (HWE – Hardy – Weinberg equilibrium) by groups. All SNPs corresponded to the Hardy-Weinberg equilibrium. Statistical analysis was carried out in the programs R statistics (Compare Groups and rstatix packages).

## Results

Such questionnaire data as alcohol consumption (p<0.001), smoking (p=0.004), consumption of animal fat (p=0.008) have significant differences in the groups and, of course, lead to the manifestation or complication of cardiovascular pathology. It is worth noting that the data demonstrate a change in the lifestyle of patients after the manifestation of pathology. Such a risk factor as heredity is well traced in the group with coronary heart disease (88%, p<0.001), while patients with stenosis noted less hereditary component (47.5%, p<0.001), which may indicate a different etiopathogenetic mechanism of pathology. Concentrations of HDL (p<0.001), LDL (p<0.001) and triglycerides (p=0.017) had significant differences in the groups, as well as coagulogram parameters (Fibrinogen (p=0.003), PTI (p<0.001), prothrombin time (p<0.001)). Such indicators can be explained by the use of hypolipidemic and anticoagulant therapy. The data is presented in table 1. 

A genetic panel for 55 polymorphisms was developed during the determining of the genetic markers associated with the risk of CHD developing in patients with stenting (second group). Information on the nucleotide sequence of the selected polymorphisms, their location in the chromosomes, genes, and genetic domain are presented in table 2. Polymorphisms associated with the risk of coronary heart disease developing are most often located on 1, 10, 7 chromosomes. The CYP2C19 gene has 7 polymorphisms in structure included in the panel. MED12L and PEAR1 genes have 3 SNPs each. The presented polymorphisms are most often located in protein-coding regions and are associated with variants of introns. The results of the statistical analysis of genotyping are presented in table 3. The results of the analysis allow us to conclude that despite the fact that all four polymorphisms have a p-value of less than 0.05, 95% CI calculated for OR indicates that only 2 polymorphisms (rs762551 and rs6785930) minor allele reduce the risk of re-stenting by 1.85 and 1.78 times, respectively. 

All polymorphisms are different, not overlapping between groups. The assessment of the association of genetic polymorphisms in the «Control vs Stenting», «Control vs CHD» groups was carried out in accordance with the case-control design based on the generalized linear model (GLM) assuming a log-additive inheritance model. 4 polymorphisms associated with the risk of CHD were identified when comparing the results of genotyping in groups of patients with coronary artery disease with control: rs762551 (p=0.019), rs12976411 (p=0.011), rs2242480 (p=0.017) and rs4977574 (p=0.02). The assumptions were applied (p-value is higher, but close to 5%) for the analysis of the results of patients with installed stent. 3 SNPs associated with stenting were identified: rs7543130 (p=0.009), rs6785930 (p=0.016), rs7819412 (p=0.061). 

## Discussion

The rs762551 polymorphism is in the CYP1A2 gene, the product of which is involved in many metabolic reactions, including methylation (R-HSA-156581), metabolic oxidation (R-HSA-211859), fatty acid metabolism (R-HSA-8978868), protectin biosynthesis (R-HSA-9018681) and much more (6). At the same time, the rs6785930 polymorphism is in the P2RY12 gene, the product of which is involved in processes related to the transmission/transfer of signals via the P2Y receptor (R-HSA-417957, R-HSA-392170) and platelet activation and aggregation (R-HSA-76002) (7,8).

It should be noted that the Bonferonni correction for multiple comparisons did not reveal significant polymorphisms associated with phenotypes (CHD, stenting, restenosis after stenting). At the same time, without taking into account the Bonferonni correction, 3 SNPs associated with stenting and 4 SNPs with coronary artery disease were detected with a small assumption (p-value is higher, but close to 5%). 

**Table 1:** Questionnaire data in patients with coronary heart disease with the absence and presence of thrombosis

Group	Control	Coronary heart disease	Thrombosis	p
Sex				<0.001
Male	42.0%	39.7%	88.6%	
Female	58.0%	60.3%	11.4%	
Age, years	51.5 [46.5;55.8]	69.0 [61.0;76.5]	62.0 [56.5;70.0]	<0.001
Circumference of the abdomen, cm	83.0 [77.2;91.5]	100 [88.0;107]	92.0 [84.0;105]	<0.001
Weight, kg	72.5 [64.2;80.0]	75.0 [65.5;82.5]	80.0 [71.5;91.0]	0.004
Height, cm	164 [159;170]	164 [160;167]	167 [165;172]	0.047
Smoking				0.004
No	78.0%	79.4%	74.3%	
Yes	22.0%	20.6%	25.7%	
Alcohol				<0.001
No	92.0%	88.9%	62.9%	
Yes	8.00%	11.1%	37.1%	
Consumption of animal fat:				0.008
No	4.00%	0.00%	5.71%	
Yes	96.0%	100%	94.3%	
Heredity of coronary heart disease				<0.001
No	64.0%	88.9%	74.3%	
Yes	36.0%	11.1%	25.7%	

**Table 2:** List of polymorphisms associated with the risk of coronary heart disease

**Rs**	**Context**	**chr**	**Symbol**	**Biotype**	**Consequence**
rs342293	TGGGCAGCCCTGTGGTTTTAATTAT[C/G]TTGAGGTTCAGGCTCACCAGTGCTC	7	—	—	—
rs4986893	ACATCAGGATTGTAAGCACCCCCTG[A/G]ATCCAGGTAAGGCCAAGTTTTTTGC	10	CYP2C19	protein_coding	stop_gained
rs56337013	CCTATGTTTGTTATTTTCAGGAAAA[C/T]GGATTTGTGTGGGAGAGGGCCTGGC	10	CYP2C19	protein_coding	missense_variant
rs1122608	TCTCATTTCGTCGTGTAAAAGGCCA[G/T]TCCCCGCCTCGCAGTGTTGGGGCAG	19	SMARCA4	protein_coding	intron_variant
rs41291556	AATCGTTTTCAGCAATGGAAAGAGA[C/T]GGAAGGAGATCCGGCGTTTCTCCCT	10	CYP2C19	protein_coding	missense_variant
rs4244285	TTCCCACTATCATTGATTATTTCCC[A/G]GGAACCCATAACAAATTACTTAAAA	10	CYP2C19	protein_coding	synonymous_variant
rs28399504	GTCTTAACAAGAGGAGAAGGCTTCA[A/G]TGGATCCTTTTGTGGTCCTTGTGCT	10	CYP2C19	protein_coding	start_lost
rs3853444	AGAGCTGACTTGTAATATGTATATA[C/T]GTTACTGGCTAATTTGATTATGGTA	4	—	—	intergenic_variant
rs3732759	TGTAGTGTAATGACTCAGTATATCT[A/G]TGGTTTATAAAAATAAATGTTTTCT	3	MED12L	protein_coding	intron_variant
rs762551	TGCTCAAAGGGTGAGCTCTGTGGGC[C/A]CAGGACGCATGGTAGATGGAGCTTA	15	CYP1A2	protein_coding	intron_variant
rs1045642	TGTTGGCCTCCTTTGCTGCCCTCAC[A/G]ATCTCTTCCTGTGACACCACCCGGC	7	ABCB1	protein_coding	synonymous_variant
rs1799853	GATGGGGAAGAGGAGCATTGAGGAC[C/T]GTGTTCAAGAGGAAGCCCGCTGCCT	10	CYP2C9	protein_coding	missense_variant
rs6787801	ATTAATGACCATATTTCAGAGCATG[A/G]TAGGGTTTTTGTTAGTGGTTTTTTG	3	MED12L	protein_coding	intron_variant
rs13431554	TTCCCATTCCTACCAAAAGGGTCAC[A/G]TCTCTCCCCGCCAAGGTCTTAATTC	2	IRS1	protein_coding	3_prime_UTR_variant
rs10306114	TAACTGAGCACCTACTACATGCTGG[A/G]CACTGCACCAGGAGATTTGTGTGCA	9	PTGS1	protein_coding	upstream_gene_variant
rs1061781	AGATCTCACGCCCAGACATCCGAAT[C/T]GGCCGCTACCGCATGATCAAGCACG	1	B4GALT2	protein_coding	synonymous_variant
rs41273215	AGGGAGACCAAAGCTGAGCTAAAGG[C/T]TTCAGTGATGCTGGGGGCTGAGAGT	1	PEAR1	protein_coding	intron_variant
rs12041331	AAGTCCCTTCTGCTGTCTCACTTCC[A/G]TCACCCTTACTCTCTGCTTTCTATA	1	PEAR1	protein_coding	intron_variant
rs1126643	ATGGTGGGGACCTCACAAACACATT[C/T]GGAGCAATTCAATATGCAAGGTAAG	5	ITGA2	protein_coding	synonymous_variant
rs1057910	TGTGGTGCACGAGGTCCAGAGATAC[C/A]TTGACCTTCTCCCCACCAGCCTGCC	10	CYP2C9	protein_coding	missense_variant
rs57731889	AGTTTCCTGGCGGCTCTGATGCCGG[C/T]CTGCCTCCTTGGCTGTCTCCCCAGG	1	PEAR1	protein_coding	intron_variant
rs1128503	GCCCACTCTGCACCTTCAGGTTCAG[A/G]CCCTTCAAGATCTACCAGGACGAGT	7	ABCB1	protein_coding	synonymous_variant
rs662	TAAACCCAAATACATCTCCCAGGAT[C/T]GTAAGTAGGGGTCAAGAAAATAGTG	7	PON1	protein_coding	missense_variant
rs6785930	TGGTGTTACCAGGCGCAGAGGTGAG[A/G]TTGTCGACGGCTTGCATTTCTTGTT	3	MED12L	protein_coding	intron_variant
rs5918	GCTCCTGTCTTACAGGCCCTGCCTC[C/T]GGGCTCACCTCGCTGTGACCTGAAG	17	ITGB3	protein_coding	missense_variant
rs2242480	CACCTCCTCCCTCCTTCTCCATGTA[C/T]CATCCACTCACCTTATTGGGTAAAA	7	CYP3A4	protein_coding	intron_variant
rs1800790	ATATAACATTACTATTGATTTTAAT[A/G]GCCCCTTTTGAAATAGAATTATGTC	4	FGB	protein_coding	upstream_gene_variant
rs2569190	AATGAAGGATGTTTCAGGGAGGGGG[A/G]CCGTAACAGGAAGGATTCTGCAGGG	5	CD14	protein_coding	upstream_gene_variant
rs1799983	CCCTGCTGCTGCAGGCCCCAGATGA[G/T]CCCCCAGAACTCTTCCTTCTGCCCC	7	NOS3	protein_coding	missense_variant
rs7543130	CACAGTCTAGACATCTCAAACTGCT[A/C]TACTATGGGGCAAGACCCCTGGACC	1	LINC01708	lncRNA	intron_variant,non_coding_transcript_variant
rs1830321	TGCATTATACTCTCTCTTTGACCTT[C/T]TAAGCATGGGAATTTTTGATTTTAT	2	TEX41	lncRNA	intron_variant,non_coding_transcript_variant
rs10455872	TCAGACACCTTGTTCTCAGAACCCA[A/G]TGTGTTTATACAGGTTAGAGGAGAA	6	LPA	protein_coding	intron_variant
rs11250135	TGCTCCCTACCCACCTCACGCCCAC[G/T]CTCCCCCAAGCAGCTTATACTGTGT	8	FAM167A	protein_coding	downstream_gene_variant
rs10406522	CCTACTCTGACACTTGAGGGCTCTC[C/T]AGCTACCCCAGTCCCAGCCATACCC	19	DOCK6	protein_coding	intron_variant
rs10861032	AAAACCCTCTATGAATTCCAATCTA[C/T]CTTTTTTAATCCGTATTTCTTCTGT	12	AC084364.3	lncRNA	non_coding_transcript_exon_variant
rs13144136	ATGTTCAACAGAGACCAACTCCCTG[G/T]CTAAAAGACAAAGCCACTGAAAGTC	4	CLNK	protein_coding	intron_variant
rs7016717	TTCTATAATCTAAATTAATGCCATC[A/G]GCTCCTAGTTAATTTTTTCATTCTC	8	—	—	intergenic_variant
rs12752401	CAGCTGGAAAAAAATGTACTAGGTT[C/T]CATTGAAATAGAGTAGTCCAATTGG	1	—	—	intergenic_variant
rs12285326	GAGATAGGAATCATGCCCTTAGCCA[A/G]GAAAGCTGTGCCTGGAACGCAGGCT	11	—	—	intergenic_variant
rs1108775	TGAAAAGGTCATATTCTGGTGGTCA[A/G]TGGCCTGCTTTAAATCTTGGCTCTA	18	—	—	intergenic_variant
rs1856746	AAGATCCCAACCTCCAGGAGAAAGC[A/G]TCATTTCACCTTTTGTTCTCCAGGC	1	FCAMR	protein_coding	intron_variant
rs2791713	CATTCCCTCCCCTACCCTGGAAAGC[A/G]AGAAAACACTCGTCGGACTGTGGGA	1	FCAMR	CTCF_binding_site	regulatory_region_variant
rs291096	GTATTCTTCCTGTATAGTTGGGATT[C/T]ACATAACCACTGGAGTCGATGACCA	1	PIGR	protein_coding	synonymous_variant
rs11012265	TTCCAGGATTCATAAAATTTCACAA[C/T]GATAGTGCTAGACTGGTCCTTGGAG	10	—	—	intergenic_variant
rs17366136	CGTCCTTGAGCCTGCAGCCGCTGCA[G/T]TGCTTTAATAACAGTTTATCCATGG	7	AC002480.1	lncRNA	upstream_gene_variant
rs13333226	GTCAAAGAGGTAGCACAGCTGTAGG[A/G]ATATTGACTCCTCTTCCCAAACAGC	16	PDILT	protein_coding	downstream_gene_variant
rs403814	CGAGGCTCGGGAGACCGGAAAGGGC[A/C]AGATGCTTCAGAGCTTGGAGGCCTT	18	L3MBTL4	protein_coding	intron_variant
rs1501908	TATCAGACCCCATTTTACAATAAAG[C/G]CATGACGCCAGAGAAGATAAAAGAC	5	HAVCR1TIMD4	—	intergenic_variant
rs6102059	CCTCTCAACAGCCCTCTGATCTACG[C/T]ACACTAACATCATGCCCATTTTACA	20	—	—	intergenic_variant
rs2271293	GCCTGGGCCCAGGGTCAATGGGGTA[A/G]AAGATGTTGATAGTGTAATGTTTGG	16	NUTF2	protein_coding	intron_variant
rs471364	GCAGAACTCCATGTGTCTGCTACCT[C/T]GCAAGCCTACCTATATTCTTTCCTC	9	TTC39B	protein_coding	intron_variant
rs174547	TGTTTTTGCTGTTTTCACCTACGCA[C/T]CCTTTTCAATAGTTGTGTTATGCTC	11	FADS1	protein_coding	intron_variant
rs7819412	AAGAAAACAACCCTAAGAAAAATCA[A/G]TCATATCAAAGCACCTTCCTTTTGT	—	XKR6	protein_coding	intron_variant
rs646776	TGGCTGATAAGCCTGTCCCTCTGCC[C/T]ATGTCCATGACACTGCTCCCAAATA	—	CELSR2	protein_coding	downstream_gene_variant
rs4977574	GGGTACATCAAATGCATTCTATAGC[A/G]CAGGATGTTCCAGTCACTCTAACAA	—	CDKN2B-AS1	lncRNA	intron_variant,non_coding_transcript_variant
rs12976411	AAAAAGTAAATAAAAATAAAAGTAT[A/T]CCTGCAAACCAGTCCAAATGCTAGT	—	ZNF507	protein_coding	downstream_gene_variant
rs7679	GGCTGCCACTGACATATGAAGATTA[C/T]GGTTCTGCCAGGGCTCCCCTCCCTG	—	ZNF335	protein_coding	downstream_gene_variant
rs12248560	AAATTTGTGTCTTCTGTTCTCAAAG[C/T]ATCTCTGATGTAAGAGATAATGCGC	—	CYP2C19	protein_coding	upstream_gene_variant
rs72552267	CGGCGTTTCTCCCTCATGACGCTGC[A/G]GAATTTTGGGATGGGGAAGAGGAGC	—	CYP2C19	protein_coding	missense_variant

**Table 3:** Polymorphisms associated with stenting and CHD

**Polymorphism**	**Group**	**Р** **-value**
**rs7543130**	Control vs Stenting	0.009
**rs6785930**	Control vs Stenting	0.016
**rs7819412**	Control vs Stenting	0.061
**rs762551**	Control vs CHD	0.019
**rs12976411**	Control vs CHD	0.011
**rs2242480**	Control vs CHD	0.017
**rs4977574**	Control vs CHD	0.02

In conclusion, four polymorphisms associated with the risk of developing coronary heart disease were revealed during the study of polymorphisms among the people of the Kazakh population: rs762551 (p=0.019), rs12976411 (p=0.011), rs2242480 (p=0.017) and rs4977574 (p=0.02). Three SNPs were determined when searching for an association with stenting due to coronary artery thrombosis: rs7543130 (p=0.009), rs6785930 (p=0.016), rs7819412 (p=0.061). It should be noted that the Bonferonni correction for multiple comparisons did not reveal significant polymorphisms associated with coronary artery disease, which requires further research with more quantity of samples.
